# The association of serum 25-hydroxyvitamin D_3_ and D_2_ with depressive symptoms in childhood – a prospective cohort study

**DOI:** 10.1111/j.1469-7610.2011.02518.x

**Published:** 2011-12-29

**Authors:** Anna-Maija Tolppanen, Adrian Sayers, William D Fraser, Glyn Lewis, Stanley Zammit, Debbie A Lawlor

**Affiliations:** 1 MRC Centre for Causal Analyses in Translational Epidemiology, School of Social and Community Medicine, University of BristolBristol; 2 School of Social and Community Medicine, University of BristolBristol; 3 Norwich Medical School, University of East AngliaNorwich; 4 MRC Centre for Neuropsychiatric Genetics and Genomics, Cardiff UniversityCardiff, UK

**Keywords:** 25-Hydroxyvitamin D, calcium, parathyroid hormone, child, depression, ALSPAC

## Abstract

**Background:**

Depression in adolescence is common and early onset predicts worse outcome in adulthood. Studies in adults have suggested a link between higher total 25-hydroxyvitamin D [25(OH)D] concentrations and lower risk of depression.

**Objectives:**

To investigate (a) the association between serum 25(OH)D_2_ and 25(OH)D_3_ concentrations and depressive symptoms in children, and (b) whether the associations of 25(OH)D_2_ and 25(OH)D_3_ are different from, and independent of, each other.

**Methods:**

Prospective cohort study with serum 25(OH)D_2_ and 25(OH)D_3_ concentrations measured at mean age of 9.8 years and depressive symptoms assessed with the Mood and Feelings Questionnaire by a trained interviewer at the mean ages of 10.6 years (*n* = 2,759) and 13.8 years (*n* = 2,752).

**Results:**

Higher concentrations of 25(OH)D_3_ assessed at mean age 9.8 years were associated with lower levels of depressive symptoms at age 13.8 years [adjusted risk ratio (RR; 95% confidence interval (CI)): 0.90 (0.86–0.95)], but not at age 10.6 years [adjusted RR (95% CI): 0.98 (0.93–1.03)] and with increased odds of decreasing symptoms between age 10.6 and 13.8 years [adjusted RR (95% CI): 1.08 (1.01–1.16)]. Serum 25(OH)D_2_ concentrations were not associated with depressive symptoms.

**Conclusions:**

This is the first study in children to suggest that the association between 25(OH)D_3_ concentrations and depression emerges in childhood. The association is independent of a wide range of potential confounding factors, and appears to be stronger with greater time separation between assessment of 25(OH)D_3_ and assessment of depressive symptoms. Confirmation of our findings in large prospective studies and trials would be valuable.

## Introduction

Depression affects 1–6% of adolescents worldwide and early onset often predicts more serious disease manifestation in later life (Thapar, Collishaw, Potter, & Thapar, 2010). Characterisation of modifiable risk factors that could be used to prevent or delay the early onset of depression is important. It has been suggested that higher concentrations of vitamin D may protect against depression in adults. Depression rates are higher in winter than in summer months, which could support a role for vitamin D (Bertone-Johnson, 2009). Some (Armstrong et al., 2007; Berk et al., 2007; Eskandari et al., 2007; Ganji, Milone, Cody, McCarty, & Wang, 2010; Hoogendijk et al., 2008; Jorde, Waterloo, Saleh, Haug, & Svartberg, 2006, Lee et al., 2010; Schneider, Weber, Frensch, Stein, & Fritz, 2000; Wilkins, Sheline, Roe, Birge, & Morris, 2006) but not all (Annweiler et al., 2010; Herran et al., 2000; Michelson et al., 1996; Nanri et al., 2009; Pan et al., 2009; Zhao, Ford, Li, & Balluz, 2010) cross-sectional studies in adults have found an association between higher serum concentrations of 25-hydroxyvitamin D (25(OH)D) and lower risk of depression in adults. Two prospective studies found that higher concentration of 25(OH)D were associated with lower risk of depression in older adults (May et al., 2010; Milaneschi et al., 2010) and three randomised controlled trials in selective populations had inconsistent findings, with a trial of vitamin D_3_ supplements improving depression symptoms in overweight/obese adults (Jorde, Sneve, Figenschau, Svartberg, & Waterloo, 2008) but two further trials of D_3_ showing no effect on symptoms in older women with seasonal affective disorder (Dumville et al., 2006) or on preventing depression in older women (Sanders et al., 2011).

25(OH)D is a robust and reliable indicator of vitamin D status, reflecting both dietary intake and synthesis in skin, which normally accounts for most of the vitamin D in humans (Seamans & Cashman, 2009). Circulatory total 25(OH)D consists of 25(OH)D_3_ [metabolite of vitamin D_3_ synthesised in skin after ultraviolet B (UVB) exposure and obtained from animal food sources] and 25(OH)D_2_ (synthesised from vitamin D_2_ obtained from plant sources). 25(OH)D_3_ and 25(OH)D_2_ are converted to 1,25-dihydroxyvitamin D_3_ and D_2_, the steroid hormones that mediate the biological actions of vitamin D. The former is known to have higher affinity to vitamin D binding protein and receptor (Glendenning et al., 2009; Houghton & Vieth, 2006) and with respect to bone health, vitamin D_3_ has been suggested to be more potent than D_2_ (Finch, Brown, & Slatopolsky, 1999). However, to date no studies have examined whether associations between these two differ with respect to depression. Vitamin D, together with parathyroid hormone (PTH), regulates calcium and phosphate homoeostasis (Brown, Dusso, & Slatopolsky, 1999; Mundy & Guise, 1999) and some (Eskandari et al., 2007; Herran et al., 2000, Hoogendijk et al., 2008; Jorde et al., 2006; May et al., 2010) but not all (Michelson et al., 1996; Schneider et al., 2000) studies have reported higher serum PTH among adults with depression. As the previous studies have included only adults, it is unknown if serum 25(OH)D concentrations are associated with mood in childhood or early adolescence. Examining this association in childhood/adolescence is important because confounding by alcohol, smoking and mood-altering drugs is somewhat less likely than in adult studies and because it is increasingly recognised that depression can emerge in childhood/adolescence (Thapar et al., 2010) and its prevention may be best started at this age.

The aims of this study were (a) to investigate the prospective association between serum 25(OH)D_2_ and 25(OH)D_3_ concentrations and depressive symptoms in children, (b) to investigate if the associations of 25(OH)D_2_ and 25(OH)D_3_ are different, and (c) to examine whether any associations were independent of serum PTH, phosphate and calcium concentrations.

## Participants and methods

### Population

The Avon Longitudinal Study of Parents and Children (ALSPAC) is a population-based birth cohort from South West England. The cohort consisted of 14,062 live births from 14,541 enrolled pregnant women who were expected to give birth between 1 April 1991 and 31 December 1992 (Golding, Pembrey, Jones and ALSPAC Study Team, 2001). From age 7, all children were invited for an annual assessment of physical and psychological development. Parents gave informed consent at enrolment, and ethical approval was obtained from the ALSPAC Law and Ethics Research Committee and the National Health Service (NHS) local research ethics committee.

**Figure 1:**
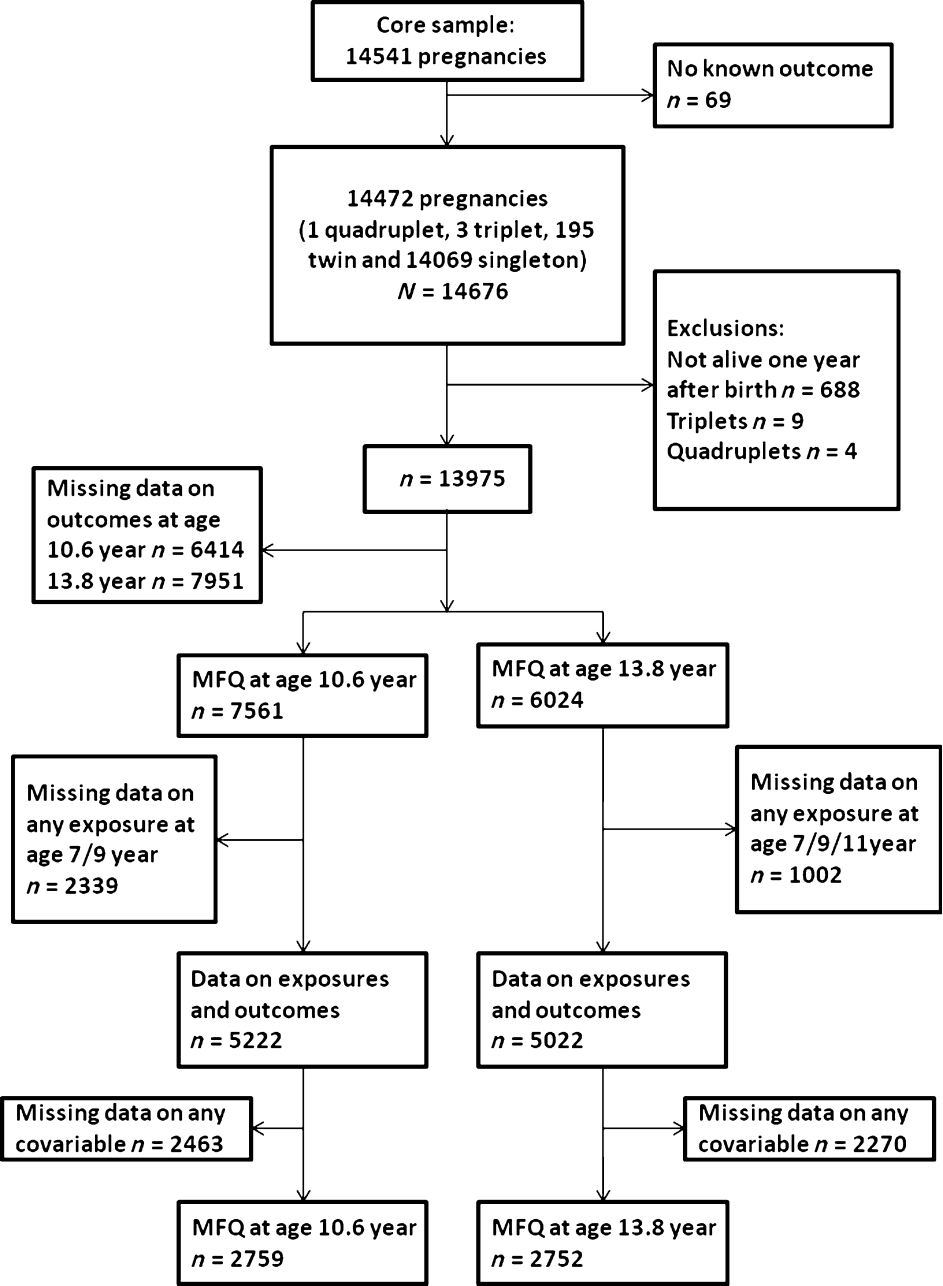
Flow of participants

Single and twin births were included in this study; the very small number of triplets and quadruplets were not included for reasons of confidentiality. Figure 1 shows how the numbers included in the analyses presented here was derived. Complete data on outcomes, exposures and confounders were available from 2,759 and 2,752 children, respectively, for assessment with outcomes at 10.6 years and with outcomes at 13.8 years.

### Outcome

The prevalence of depressive symptoms was evaluated with the Mood and Feelings Questionnaire (MFQ) by a trained interviewer at the mean ages of 10.6 and 13.8 years. The MFQ is a 13-item depression inventory validated for use in 6- to 18-year-olds. Each item is scored between 0 and 2, resulting to a maximum score of 26 with higher scores indicating presence of symptoms of depression. MFQ correlates highly with more extensive evaluations like the Children’s Depression Inventory and the Diagnostic Interview Schedule for Children (Costello & Angold, 1988).

The MFQ scores were positively skewed even after various transformations so we generated three categories of the score (0–2, 3–5, ≥ 6), representing approximate thirds of the distribution. An ordinal categorical variable was derived to indicate change in MFQ score category (increase by two categories/increase by one category/no change/decrease by one category/decrease by two categories) between ages 10.6 and 13.8 years.

### Exposures and phlebotomy-based covariables

Serum 25(OH)D_3_, 25(OH)D_2_, PTH, phosphate and calcium were assayed on nonfasting blood samples collected at mean age 9.9 years for the majority of participants (*n* = 2,130 for MFQ assessed at age 13.8 years and *n* = 2,493 for MFQ assessed at age 10.6 years). If no samples were available from the 9.9-year assessment, samples from mean age 11.8 years (*n* = 416) or, second, the 7.6-year assessments (*n* = 206 for MFQ assessed at age 13.8 years and *n* = 266 for MFQ assessed at age 10.6 years) were used. The mean age at sample collection in the whole study sample was 9.8 years (standard deviation: *SD* = 0.74). To keep the analyses prospective, we excluded exposure measurements that were taken at 11.8-year clinic when the outcome was measured at age 10.6 years.

Following collection, samples were immediately spun, frozen and stored at −80 °C. Assays were performed after a maximum of 12 years in storage with no previous freeze-thaw cycles. 25(OH)D_3_, 25(OH)D_2_ and deuterated internal standard were extracted from serum samples, following protein precipitation, using Isolute C18 solid phase extraction cartridges. Potential interfering compounds were removed by initial elution with 50% methanol followed by elution of the vitamins using 10% tetrahydrofuran in acetonitrile. Dried extracts were reconstituted prior to injection into a high-pressure liquid chromatography tandem mass spectrometer (Waters Acuity, Manchester, UK). The following transitions (mass to charge ratio) in multiple reaction mode were used: 413.2 > 395.3, 401.1 > 383.3 and 407.5 > 107.2 for 25(OH)D_2_, 25(OH)D_3_, and hexa-deuterated 25(OH)D_3_ respectively. Interassay coefficients of variation for the assay were < 10% across a working range of 1–250 ng/ml for both 25(OH)D_3_ and 25(OH)D_2_. Measurements were performed in a laboratory meeting the performance target set by the Vitamin D External Quality Assessment Scheme (DEQAS) Advisory Panel for 25(OH)D assays.

Total serum calcium, phosphate and albumin concentrations were measured by standard laboratory methods on Roche Modular analysers (Roche Diagnostics Ltd, West Sussex, UK). Serum calcium was adjusted for albumin using a normogram of calcium and albumin distributions of the samples analysed in the clinical chemistry laboratory where the measurements were performed and albumin-adjusted calcium was used in all statistical analyses. Intact parathyroid hormone [iPTH(1–84)] was measured by electrochemiluminescent immunoassay on Elecsys 2010 immunoanalyzer (Roche, Lewes, UK). Interassay coefficient of variation was < 6% from 2 to 50 pmol/L. The assay sensitivity was 1 pmol/L.

### Confounding factors

We considered gender, age, ethnicity (white, nonwhite), head of household occupational social class, maternal and paternal education, family history of depression or schizophrenia, UVB exposure, body mass index (BMI) and cognitive function to be important confounders because of their known associations with 25(OH)D_3_ concentrations and depressive symptoms. We also adjusted for pubertal stage as this might affect depressive symptoms and 25(OH)D_3_. Data on head of household occupational social class, ethnicity, parents’ education and family history of depression and schizophrenia were obtained from parent-completed questionnaires. Time spent outdoors during summer months on school days, school weekends and holidays was reported as *None*, *1 hr/day*, *1–2 hr/day* and *3 or more hr/day* in parent-completed questionnaires at mean age of 8.5 years. Responses were coded as follows: *None* = 0, *1* = 1, *1–2* = 1.5 and *3* = 5. Average hours spent outdoors per summer day (1 June–31 August) were calculated using term dates from Bristol City Council’s Education Committee term dates for 2001–2002 (summer term 1 June–24 July, holidays 24 July–31 August). Information on protection from UVB exposure (use of sunblock, covering clothing or hat and avoidance of midday sun) were obtained from the same questionnaires. A summary variable for UVB protection score was derived by scoring the responses to questions on use of sunblock, covering clothing or hat and avoidance of midday sun as *Always* = 3, *Usually* = 2, *Sometimes* = 1, *Never* = 0 and summing these scores. This gives a single variable that ranges from 0 to 12, with 0 indicating the least meticulous protection from UVB.

Height and weight were measured at the same time as blood samples for obtaining 25(OH)D_3_ and other assays and were used to calculate BMI. Total IQ score in Wechsler Intelligence Scale for Children (WISC–III UK version) was assessed at mean age 8.5. Puberty stage was assessed by parental report using Tanner staging (Tanner, 1962) of pubic hair, breast and genitalia development on repeat occasions. In our analyses, we used data from the questionnaire closest to the time of phlebotomy for the exposures for each child.

**Figure 2:**
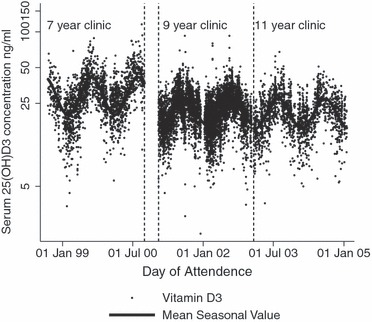
Seasonal variation in serum 25(OH)D_3_ concentrations

25(OH)D_3_ concentrations had displayed a sinusoidal seasonal variation (Figure 2). In order to derive a value of 25(OH)D_3_ for each participant that was accurately adjusted for the seasonal effects of when the sample was taken, we used linear regression with date of blood sampling as the independent variable and log_e_ 25(OH)D_3_ as the outcome (dependent) variable and with trigonometric sine and cosine functions. 25(OH)D_3_ was log_e_ transformed in this regression model to ensure that the residuals in the sine–cosine regression were approximately normally distributed. The residuals from this regression are the participants logged 25(OH)D_3_ having adjusted for seasonal differences in when the sample was taken. This seasonal adjusted 25(OH)D_3_ variable represents a participants average 25(OH)D_3_ across seasons and is the main 25(OH)D_3_ exposure measurement used in our analyses [we also present associations without this adjustment]. There was no strong seasonal variation in 25(OH)D_2_ concentrations.

### Statistical analyses

Statistical analyses were conducted with Stata 11.0 (Stata Corp LP, College Station, TX).

To include all participants on whom a 25(OH)D_2_ was assayed, those with a value below the detectable limit of the assay (0.5 ng/ml) were given a value of 0.5 ng/ml and indicated using a binary covariable in all regression models. Serum 25(OH)D_3_, 25(OH)D_2,_ calcium, phosphate and PTH concentrations were age- and gender-standardised using the internal cohort data with age in 1-month categories.

The association of potential confounders with serum 25(OH)D_3_, 25(OH)D_2,_ calcium, phosphate and PTH concentrations was assessed with linear regression and associations of confounders with depressive symptoms with ordered logistic regression. We investigated the linearity of associations between 25(OH)D_3_ and 25(OH)D_2_ and depressive symptoms by splitting the 25(OH)D variables into fifths of their distribution and graphically examining the odds of depressive symptoms across these fifths. We statistically tested for linearity by obtaining a *p* value for linear trend from a regression model in which these fifths were entered as a continuous score (1–5). Deviation from linearity was assessed with a likelihood ratio test comparing regression models in which the fifths were entered as categories (four indicator variables) and one in which they were entered as a continuous score.

The main analyses assessing associations of seasonally adjusted 25(OH)D_3_ and 25(OH)D_2_ with outcomes were done with a nonparametric bootstrap procedure (10,000 replications) in conjunction with ordered logistic regression using bsample and ologit commands in Stata. The bootstrapping procedure (Efron & Tibshirani, 1986) enabled us to statistically compare associations of 25(OH)D_3_ to those of 25(OH)D_2_. The difference between the effect of 25(OH)D_3_ and 25(OH)D_2_ was calculated from the bootstrap replicate distribution To numerically compare the associations of two forms of 25(OH)D, we scaled them the same by multiplying the beta coefficients from the regression models by log_e_(2). The results are interpreted as the difference in odds of MFQ between the lowest and middle or middle and highest MFQ score per doubling of exposure.

In addition to examining associations with 25(OH)D_2_ and 25(OH)D_3_ on a continuous scale, we also examined the association between total 25(OH)D deficiency and insufficiency and depressive symptoms. Total 25(OH)D was calculated by summing 25(OH)D_2_ and 25(OH)D_3_ and insufficiency was defined as a value below 30 ng/ml and deficiency below 20 ng/ml (Holick, 2007). Finally, in a sensitivity analysis we examined the association of 25(OH)D_3_ that was not adjusted for seasonal variation with outcomes. All association analyses were performed for both genders combined as there was no strong statistical evidence for Gender × Exposure interaction (*p* ≥ .20).

## Results

The mean (interquartile range) serum concentrations of season-adjusted 25(OH)D_3_ and 25(OH)D_2_ were 24.9 (24.7–25.1) and 1.3 (0.5–2.7) ng/ml, respectively. The median (interquartile range) of serum phosphate, calcium and PTH were 1.54 (1.43–1.64) mmol/L, 2.38 (2.31–2.44) mmol/L and 4.5 (3.4–5.8) pmol/L respectively. Other characteristics of the participants are shown in Table S1.

Table S2 shows the associations of 25(OH)D_3_, 25(OH)D_2_ phosphate, calcium and PTH concentrations with potential confounders. Those of nonwhite ethnicity had higher PTH and lower 25(OH)D_3_ concentrations. BMI was negatively associated with serum 25(OH)D_3_ and 25(OH)D_2_ concentrations and positively with PTH concentrations. Higher socioeconomic position of parents was associated with lower concentrations of calcium and 25(OH)D_2_ and higher concentrations of 25(OH)D_3_. Less meticulous protection from UVB was associated with lower PTH concentrations and higher 25(OH)D_3_ concentrations. Children who spent more time outdoors during summer had higher 25(OH)D_3_ and 25(OH)D_2_ concentrations. Children with family history of psychiatric problems had lower 25(OH)D_3_ and calcium concentrations. Those with more advanced puberty stage had lower 25(OH)D_3_ concentrations and higher calcium concentrations.

Table S3 summarises the associations of confounders and depressive symptoms at ages 10.6 and 13.8 years. Higher socioeconomic position and IQ were associated with lower risk of depressive symptoms at age 10.6 years and higher BMI with higher risk of depressive symptoms at age 13 years. Children who spent more time outdoors during summer had lower risk of depressive symptoms at age 13.8 years. Children with family history of psychiatric problems had higher risk of depressive symptoms at both ages.

**Figure 3:**
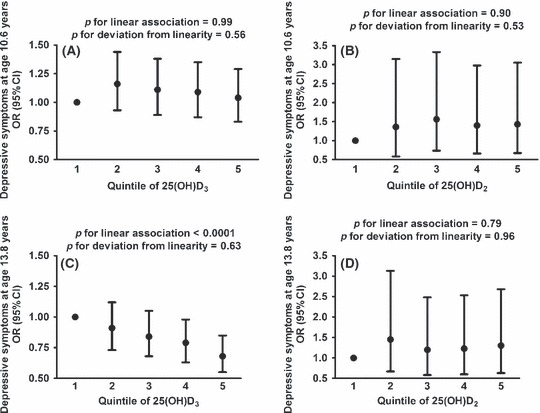
Odds of depressive symptoms at age 10.6 years (A and B) and age 13.8 years (C and D) by fifths of the distribution of 25(OH)D_3_ (A and C) and 25(OH)D_2_ (B and D)

The associations of 25(OH)D_3_ and 25(OH)D_2_ with depressive symptoms were either linear or null, with no strong evidence of deviation from linearity or suggestion of a threshold association (Figure 3). Table 1 shows the prospective associations between serum 25(OH)D_3_, 25(OH)D_2_, phosphate, calcium and PTH concentrations and depressive symptoms at ages 10.6 and 13.8 years. Higher concentrations of 25(OH)D_3_ were associated with lower risk of depressive symptoms at age 13.8 years but not at age 10.6 years. 25(OH)D_2_ was not associated with depressive symptoms at either age. There was statistical evidence that the association of 25(OH)D_3_ with depressive symptoms at age 13.8 years differed from the association of 25(OH)D_2_ with the same outcome (*p* ≤ .001), but there was no evidence of difference in associations of 25(OH)D_3_ and 25(OH)D_2_ with symptoms at age 10.6 years (*p* = .81). There was some suggestion that higher concentrations of PTH were associated with lower risk of depressive symptoms at age 13.8 years after adjusting for all confounders and other measured analytes, but confidence intervals were wide and included the null value. Other exposures were not associated with depressive symptoms.

**Table 1:** Prospective association of 25(OH)D_3_, 25(OH)D_2_, phosphate, albumin-adjusted calcium and parathyroid hormone concentrations with depressive symptoms assessed by short Mood and Feelings Questionnaire at age 10.6 years (*n* = 2,759; exposures assessed at mean age 9.2 years) and age 13.8 years (*n* = 2,752; exposures assessed at mean age 9.8 years)

		OR for category change per doubling of exposure (95% CI)		
		
Outcome	Exposure	Model 1^a^	Model 2^b^	Model 3^c^
Depressive symptoms at age 10.6 years	25(OH)D_3_	0.98 (0.93, 1.03)	0.98 (0.93, 1.03)	0.98 (0.93, 1.03)
	25(OH)D_2_	1.02 (0.94, 1.10)	1.01 (0.93, 1.10)	0.99 (0.94, 1.04)
	Albumin-adjusted calcium	1.02 (0.97, 1.07)	1.01 (0.97, 1.06)	1.00 (0.95, 1.05)
	Phosphate	1.03 (0.98, 1.08)	1.04 (0.99, 1.09)	1.04 (0.98, 1.09)
	Parathyroid hormone	0.98 (0.93, 1.03)	0.98 (0.94, 1.03)	0.97 (0.93, 1.03)
Depressive symptoms at age 13.8 years	25(OH)D_3_	0.90 (0.86, 0.94)	0.91 (0.86, 0.95)	0.90 (0.86, 0.95)
	25(OH)D_2_	1.02 (0.95, 1.11)	1.03 (0.95, 1.12)	1.02 (0.97, 1.08)
	Albumin-adjusted calcium	0.99 (0.94, 1.04)	1.00 (0.95, 1.04)	0.99 (0.94, 1.04)
	Phosphate	1.01 (0.97, 1.06)	1.02 (0.97, 1.06)	1.02 (0.98, 1.07)
	Parathyroid hormone	0.99 (0.94, 1.03)	0.98 (0.93, 1.02)	0.96 (0.91, 1.01)

OR, odds ratio.

a Model 1 is unadjusted (the exposures are standardised for age and gender and 25OHD_3_ is adjusted for season and ethnicity).

b Model 2 is adjusted for ethnicity, head of household social class, mothers and partners education, time spent outdoors during summer (age 8.5 years), UVB protection score, WISC IQ score at 8.5 years, BMI, family history of psychiatric problems and puberty stage.

c Model 3 is adjusted for Model 2 plus serum concentrations of other hormones/metabolites which are related vitamin D homeostasis [e.g. association of 25(OH)D_3_ is adjusted for 25(OH)D_2_, phosphate, albumin-adjusted calcium and parathyroid hormone].

The associations of 25(OH)D_3_ concentrations which were not adjusted for seasonality (Table S4) and of total 25(OH)D (Table S5) were identical to those of seasonal adjusted 25(OH)D_3_. Consistent with these findings depressive symptoms at age 10.6 years did not differ between those with total 25(OH)D deficiency or total 25(OH)D insufficiency, whereas those with either deficiency or insufficiency had increased risk of depressive symptoms of 20–30% at age 13.8 (Table 2).

**Table 2:** Prospective association of total 25(OH)D deficiency and insufficiency with depressive symptoms assessed by short Mood and Feelings Questionnaire at age 10.6 years (*n* = 2,759; vitamin D status assessed at mean age 9.2 years) and age 13.8 years (*n* = 2,752; vitamin D status assessed at mean age 9.8 years)

		OR for category decrease per doubling of exposure (95% CI)		
		
Outcome	Exposure	Model 1^a^	Model 2^b^	Model 3^c^
Depressive symptoms at age 10.6 years	Vitamin D deficiency	0.97 (0.87, 1.08)	0.97 (0.88, 1.09)	0.98 (0.87, 1.09)
	Vitamin D insufficiency	1.00 (0.90, 1.11)	1.02 (0.91, 1.16)	1.01 (0.90, 1.13)
Depressive symptoms at age 13.8 years	Vitamin D deficiency	1.20 (1.08, 1.33)	1.19 (1.07, 1.33)	1.21 (1.08, 1.36)
	Vitamin D insufficiency	1.26 (1.12, 1.42)	1.26 (1.13, 1.41)	1.27 (1.12, 1.42)

OR, odds ratio.

a Model 1 is unadjusted.

b Model 2 is adjusted for ethnicity, age, gender, head of household social class, mothers and partners education, time spent outdoors during summer (age 8.5 years), UVB protection score, WISC IQ score at 8.5 years, BMI, maternal history of psychiatric problems and puberty stage.

c Model 3 is adjusted for Model 2 plus serum concentrations of phosphate, albumin-adjusted calcium and parathyroid hormone.

Table 3 shows change in depressive symptoms between age 10.6 and 13.8 years. We present these for all participants with valid responses to the MFQ at both ages (*N* = 4,487) and also those with complete data on MFQ at both ages and all covariables used in any multivariable models (*N* = 1,815). The distributions of the change variables are very similar in these two groups. The most common occurrence was for participants to remain stable over time or for depressive symptoms to have increased by one category. The least common was for a decrease in depressive symptoms by two categories.

**Table 3:** Changes in depressive symptom categories assessed by change in Mood and Feelings Questionnaire (MFQ) score category between age 10.6 and 13.8 years (in all participants with MFQ data at both ages (*N* = 4,487) and those with complete data on MFQ at both ages and on all other variables included in any multivariable analyses (*N* = 1,815)

Category	All participants with valid MFQ data at both ages, *n* (%)	Participants with complete data on MFQ at both ages and all other covariables, *n* (%)
Increase by two categories	582 (13.0)	247 (13.6)
Increase by one category	1,285 (28.7)	534 (29.4)
No change	1,285 (28.7)	520 (28.7)
Decrease by one category	1,037 (23.1)	421 (23.2)
Decrease by two categories	298 (6.6)	93 (5.2)

Table 4 shows the associations of serum concentrations of 25(OH)D_3,_ 25(OH)D_2,_ calcium, phosphate and PTH with changes in depressive symptom category. A doubling in 25(OH)D_3_ concentrations was associated with a 9% increased odds of depressive symptoms decreasing between age 10.6 and 13.8 years, with little effect of adjustment for potential confounding factors. 25(OH)D_2_, calcium, phosphate or PTH were not associated with change in depressive symptoms over time and there was statistical evidence of a different association of 25(OH)D_3_ and 25(OH)D_2_ with change in depressive symptoms (*p* = .012).

**Table 4:** Prospective association of 25(OH)D_3,_ 25(OH)D_2_, phosphate, albumin-adjusted calcium and parathyroid hormone concentrations (assessed at mean age 9.2 years) with decrease in depressive symptoms [*N* = 1,815; five categories: increase by two categories (reference); increase by one category; no change, decrease by one category: decrease by two categories]

	OR for category decrease per doubling of exposure (95% CI)		
	
Exposure	Model 1^a^	Model 2^b^	Model 3^c^
25(OH)D_3_	1.09 (1.02, 1.16)	1.08 (1.01, 1.16)	1.08 (1.01, 1.16)
25(OH)D_2_	0.97 (0.88, 1.07)	0.96 (0.86, 1.06)	0.95 (0.90, 1.01)
Albumin-adjusted calcium	1.03 (0.97, 1.09)	1.01 (0.96, 1.08)	1.01 (0.96, 1.08)
Phosphate	0.99 (0.94, 1.05)	0.99 (0.94, 1.05)	0.99 (0.93, 1.05)
Parathyroid hormone	0.97 (0.92, 1.03)	0.98 (0.93, 1.04)	0.99 (0.93, 1.05)

OR, odds ratio.

a Model 1 is unadjusted (the exposures are standardised for age and gender and 25OHD_3_ is adjusted for season and ethnicity).

b Model 2 is adjusted for ethnicity, head of household social class, mothers and partners education, time spent outdoors during summer (age 8.5 years), UVB protection score, WISC IQ score at 8.5 years, BMI, maternal history of psychiatric problems and puberty stage.

c Model 3 is adjusted for Model 2 plus serum concentrations of other hormones/metabolites which are related vitamin D homeostasis (e.g. association of 25(OH)D_3_ is adjusted for 25(OH)D_2,_ phosphate, albumin-adjusted calcium and parathyroid hormone.

## Discussion

In our prospective study of children, we have found higher concentrations of season-adjusted 25(OH)D_3_ assessed at mean age 9.8 years to be associated with lower levels of depressive symptoms at age 13.8 years and with increased odds of decreasing symptoms between age 10.6 and 13.8 years. These associations were independent of a wide range of potential confounders, as well as of 25(OH)D_2_, calcium, phosphate and PTH concentrations, which were not strongly associated with depressive symptoms at either age. We also found statistical evidence that the association of 25(OH)D_3_ with depressive symptoms was stronger than that of 25(OH)D_2_. The association of 25(OH)D_3_ with depressive symptoms was linear across the distribution of 25(OH)D_3_ concentrations, suggesting that even amongst those with what would be considered normal concentrations, an increase might result in somewhat lower depressive symptoms (if our findings are in future studies shown to represent causal effects). Consistent with these findings, and reflecting the fact that 25(OH)D_3_ is the biggest contributor to total 25(OH)D, we found that risk of depressive symptoms was greater at 13.8 years in those with total 25(OH)D deficiency or total 25(OH)D insufficiency.

To our knowledge, this is the first prospective study to examine this association in children. Our findings in this cohort of children are consistent with findings from the two prospective studies in adults (May et al., 2010; Milaneschi et al., 2010) and from one randomised controlled trial that examined the effect of vitamin D_3_ supplementation on depressive symptoms in adults (Jorde et al., 2008).

The association of 25(OH)D_3_ with depressive symptoms in children only emerged with symptoms measured 3 years after exposure assessment, and was not present when symptoms were assessed just 1 year after exposure assessment. One might expect a stronger association with the earlier age, possibly in part because of reverse causality [i.e. depressive symptoms resulting in less outdoor activity and hence reduced vitamin 25(OH)D_3_ concentrations]. It is possible that children with higher concentrations of 25(OH)D_3_ at age 9.8 years have on average higher concentrations over the subsequent 3 years and that the inverse association with depressive symptoms requires an accumulation of consistent concentrations. Alternatively, factors other than 25(OH)D_3_ but that are associated with it and accumulate over time (e.g. outdoor physical activity) might explain the association. The risk factors for childhood–onset depression may also be different from those of adolescence–onset depression (Jaffee et al., 2002). Lastly, it is possible that the biological pathways linking 25(OH)D_3_ to depression involve a chain of effects that take some time to emerge.

The stronger association of 25(OH)D_3_ compared to 25(OH)D_2_ could be a chance finding. The difference could also reflect possible greater residual confounding by, for example, different dietary patterns associated with 25(OH)D_2_ and 25(OH)D_3_ or outdoor physical activity, which will affect D_3_ more than D_2_; whilst we have attempted to adjust for a wide range of potential confounding factors residual confounding is possible. Lastly, the different associations could be explained by D_3_ being truly more potent at preventing depressive symptoms than D_2_. This finding requires further replication in other studies before we can conclude that D_3_ is more strongly associated with depressive symptoms than is D_2_.

Vitamin D receptors are expressed throughout the brain and both 25(OH)D_3_ and 25(OH)D_2_ cross the blood–brain barrier (Eyles, Smith, Kinobe, Hewison, & McGrath, 2005). Animal studies have shown that vitamin D is essential for normal neurogenesis (Cui, McGrath, Burne, Mackay-Sim, & Eyles, 2007), learning ability and behaviour in rodents (Becker, Eyles, McGrath, & Grecksch, 2005; Eyles et al., 2006; Harms, Eyles, McGrath, Mackay-Sim, & Burne, 2008), but currently it is unknown if the neural actions of vitamin D metabolites affect monoamine concentrations, hypothalamic–pituitary–adrenal axis responsiveness to stress or other mechanisms involved in depression (Belmaker & Agam, 2008).

Contrary to previous studies showing an association between higher PTH concentrations and depression in adults (Hoogendijk et al., 2008; May et al., 2010), serum concentrations of PTH were not strongly associated with depressive symptoms in children in our study. The differences between our results and these previous studies in adults could be explained by differences in outcome measurement and study sample or could reflect real differences in these associations by age.

### Study strengths and limitations

To our knowledge, this is the first study to examine these associations in children and is one of the few studies to examine them prospectively. We had a large sample size and were able to examine potential confounding by a wide range of characteristics and study the different effects of 25(OH)D_3_ and 25(OH)D_2_. We also used self-reported, rather than parent-reported depressive symptoms. This is important because parent-reported scores do not reveal depressive symptoms as early as self-reported measures (Cole et al., 2002). Consistent with other prospective cohort studies there has been substantial attrition over time with those who continued to attend the follow-up clinics being more likely to be from higher socioeconomic backgrounds (Golding, Pembrey, Jones and ALSPAC Study Team, 2001). Twenty-seven per cent of our study population had total 25(OH)D concentration below 20 ng/ml so the results are likely to apply to other populations with high prevalence of low 25(OH)D concentrations (Lips, 2010; Mansbach, Ginde, & Camargo, 2009).

Depressive symptoms were analysed as a categorical variable instead of a continuous score. This might have lost some refinement, but this was necessary due to highly skewed distribution. Serum 25(OH)D_3_, D_2_, phosphate, calcium and PTH were measured on a single occasion and this may not accurately reflect usual status (Schram et al., 2007). However, previous epidemiological studies in adults (including with bone phenotypes for which these exposures have established biological relationships) also use single measurements and for season-specific vitamin D status over a longer time, a single measure is likely to be adequate (Hofmann, Yu, Horst, Hayes, & Purdue, 2010) and serum calcium concentrations are normally maintained within relatively narrow limits in humans (Parfitt, 1987). As noted above, our results do not imply causality and the association of 25(OH)D_3_ with depressive symptoms 3 years later might be explained by residual confounding.

## Conclusions

Our results suggest that the association between 25(OH)D_3_ concentrations and depression emerges in childhood. The association is independent of a wide range of potential confounding factors, and appears to be stronger with greater time separation between assessment of 25(OH)D_3_ and that of depressive symptoms. Given the importance of depression in childhood and adolescence and the relative ease with which 25(OH)D_3_ could be increased through supplementation, randomised controlled trials to assess the effectiveness of this for prevention of depressive symptoms in this age group would be appropriate.

**Key points**

- Depression in adolescence is common and early onset predicts worse outcome in adulthood.
- Previous, mainly cross-sectional studies in adults have suggested a link between higher total 25-hydroxyvitamin D [25(OH)D] concentrations and lower risk of depression.
- Findings from this first prospective study in children suggest that the linear association between 25(OH)D concentrations and depression emerges in childhood/early adolescence and is driven by the 25(OH)D_3_ form.
- 25(OH)D_2_ concentrations were not associated with depressive symptoms.

## Supporting information

**Table S1** Sample characteristics (only complete cases for depressive symptoms at age 13.8 years are included)

**Table S2** Univariable associations between potential confounders and age- and gender-standardised serum 25-hydroxyvitamin D_3_, D_2_, phosphate, calcium and PTH concentrations

**Table S3** Univariable associations between potential confounders, exposures and depressive symptoms

**Table S4** Prospective association of unadjusted 25(OH)D_3_ with depressive symptoms assessed by short Moodand Feelings Questionnaire at age 10.6 years (*n* = 2,759; exposures assessed at mean age 9.2 years) and age 13.8 years (*n* = 2,752; exposures assessed at mean age 9.8 years)

**Table S5** Prospective association of total 25(OH)D [25(OH)D_3_ + 25(OH)D_2_] with depressive symptoms assessed by short Mood and Feelings Questionnaire at age 10.6 years (*n* = 2,759; exposures assessed at mean age 9.2 years) and age 13.8 years (*n* = 2,752; exposures assessed at mean age 9.8 years)
