# Supplementary material for: The association of serum 25-hydroxyvitamin D3 and D2 with depressive symptoms in childhood – a prospective cohort study
Source: J Child Psychol Psychiatry. 2011 Dec 29;53(7):757–66. doi: 10.1111/j.1469-7610.2011.02518.x (PMC3412227; doi:10.1111/j.1469-7610.2011.02518.x)
Supplement: Supplementary file 1 [file jcpp0053-0757-SD1.doc]

**Online Supplementary Table 1.** Sample characteristics (only complete cases for depressive symptoms at age 13.8 years are included)

|  |  | Mean (Interquartile range) | Range |
| --- | --- | --- | --- |
| Age (years) |  | 9.80 (9.58-10.10) | 7.25-12.75 |
| BMI (kg/m2) |  | 17.58 (15.62-18.88) | 12.44-35.06 |
| WISC full IQ score at 8.5 years |  | 108.06 (97-119) | 49-148 |
| UVB protection score |  | 7.41 (6-9) | 0-12 |
| Average h/day spent outdoors during summer |  | 3.97 (3.61-4.95) | 0.99-5.00 |
| MFQ score at age 10.6 |  | 3 (1-5) | 0-21 |
| MFQ score at age 13.8 |  | 4 (2-7) | 0-25 |
|  |  |  |  |
|  | Category | n (%) |  |
| Ethnicity | White | 2682 (97.5) |  |
|  | Non-white | 70 (2.5) |  |
| Gender | Boy | 1293 (47.0) |  |
|  | Girl | 1459 (53.0) |  |
| Head of household social class | i | 539 (19.6) |  |
|  | ii | 1340 (48.7) |  |
|  | iii non-manual | 611 (22.2) |  |
|  | iii manual | 192 (7.0) |  |
|  | iv/v | 70 (2.5) |  |
| Paternal education | None/CSE | 355 (12.9) |  |
|  | Vocational | 189 (6.9) |  |
|  | O level | 561 (20.4) |  |
|  | A level | 880 (32.0) |  |
|  | Degree | 767 (27.9) |  |
| Maternal education | None/CSE | 211 (7.7) |  |
|  | Vocational | 178 (6.5) |  |
|  | O level | 953 (34.6) |  |
|  | A level | 814 (29.6) |  |
|  | Degree | 596 (21.7) |  |
| Family history of depression/schizophrenia | None | 2062 (74.9) |  |
|  | Depression | 649 (23.6) |  |
|  | Schizophrenia | 41 (1.5) |  |
| Puberty stage at serum measurement | 1 | 1192 (43.3) |  |
|  | 2 | 895 (32.5) |  |
|  | 3 | 512 (18.6) |  |
|  | 4-5 | 153 (5.5) |  |
| Vitamin D deficiency  (total 25(OH)D<20ng/mL) | No | 1990 (72.3) |  |
|  | Yes | 769 (27.7) |  |
| Vitamin D insufficiency  (total 25(OH)D<30ng/mL) | No | 621 (22.5) |  |
|  | Yes | 2138 (77.5) |  |

**Online Supplementary Table 2. Univariable associations between potential confounders and age and gender standardised serum 25-hydroxyvitamin D_3_, D_2_, phosphate, calcium and PTH concentrations**

|  | Season-adjusted 25(OH)D_3_ | | 25(OH)D_2_ | | Phosphate | | Albumin-adjusted calcium | | | Parathyroid hormone | |
| --- | --- | --- | --- | --- | --- | --- | --- | --- | --- | --- | --- |
|  | SD change per SD/category change (95%CI) | *P* | SD change per SD/category change (95%CI) | *P* | SD change per SD/category change (95%CI) | *P* | SD change per SD/category change (95%CI) | | *P* | SD change per SD/category change (95%CI) | *P* |
| BMI (kg/m^2^) | -0.02  (-0.03, -0.01) | <0.001 | -0.01  (-0.02, 0.00) | 0.002 | 0.00  (-0.01, 0.01) | 0.68 | 0.00  (-0.01 to 0.01) | | 0.99 | 0.02  (0.01 to 0.03) | <0.001 |
| WISC full IQ score at 8.5 years | 0.00  (0.00, 0.00) | 0.17 | -0.01  (-0.01, -0.01) | 0.004 | 0.00  (0.00, 0.00) | 0.26 | 0.00  (0.00 to 0.00) | | 0.01 | 0.00  (0.00 to 0.00) | 0.76 |
| Non-white ethnicity | -0.67  (-0.78, -0.57) | <0.001 | -0.08  (-0.19, 0.03) | 0.13 | 0.02  (-0.09, 0.12) | 0.78 | -0.02  (-0.12 to 0.09) | | 0.78 | 0.35  (0.24 to 0.45) | <0.001 |
| Head of household social class |  |  |  |  |  |  |  | |  |  |  |
| i | reference | 0.002 | reference | <0.001 | reference | 0.08 | reference | | 0.11 | reference | 0.55 |
| ii | 0.03  (-0.04, 0.10) |  | 0.04  (-0.03, 0.11) |  | 0.00  (-0.07, 0.07) |  | 0.05  (-0.02, 0.13) | |  | 0.02  (-0.05, 0.09) |  |
| iii non-manual | 0.00  (-0.08, 0.08) |  | 0.06  (-0.02, 0.14) |  | -0.06  (-0.14, 0.02) |  | 0.10  (0.02, 0.18) | |  | -0.04  (-0.12, 0.04) |  |
| iii manual | -0.12  (-0.21, -0.03) |  | 0.18  (0.09, 0.28) |  | -0.07  (-0.17, 0.02) |  | 0.03  (-0.06, 0.13) | |  | 0.06  (-0.03, 0.16) |  |
| iv/v | -0.12  (-0.25, 0.01) |  | 0.14  (0.00, 0.27) |  | -0.01  (-0.14, 0.12) |  | 0.11  (-0.03, 0.24) | |  | 0.06  (-0.07, 0.19) |  |
| Paternal education |  |  |  |  |  |  |  |  | |  |  |
| None/CSE | reference | 0.01 | reference | 0.001 | reference | 0.42 | reference | <0.001 | | reference | 0.62 |
| Vocational | 0.11  (0.01, 0.20) |  | -0.15  (-0.25, -0.05) |  | 0.00  (-0.10, 0.10) |  | -0.08  (-0.18, 0.01) |  | | -0.02  (-0.12, 0.08) |  |
| O level | 0.12  (0.05, 0.20) |  | 0.00  (-0.08, 0.08) |  | 0.01  (-0.07, 0.08) |  | -0.06  (-0.14, 0.01) |  | | 0.02  (-0.06, 0.09) |  |
| A level | 0.14  (0.07, 0.21) |  | -0.08  (-0.15, -0.01) |  | -0.01  (-0.08, 0.06) |  | -0.07  (-0.14, 0.00) |  | | 0.14  (0.06, 0.21) |  |
| Degree | 0.08  (0.01, 0.15) |  | -0.15  (-0.22, -0.07) |  | 0.04  (-0.03, 0.12) |  | -0.16  (-0.23, -0.08) |  | | -0.02  (-0.09, 0.06) |  |
| Maternal education |  |  |  |  |  |  |  |  | |  |  |
| None/CSE | reference | 0.25 | reference | <0.001 | reference | 0.11 | reference | <0.001 | | reference | 0.71 |
| Vocational | 0.12  (0.01, 0.22) |  | -0.11  (-0.21, 0.00) |  | 0.09  (-0.02, 0.19) |  | 0.02  (-0.09, 0.12) |  | | -0.11  (-0.22, -0.01) |  |
| O level | 0.14  (0.06, 0.21) |  | -0.07  (-0.15, 0.00) |  | -0.01  (-0.09, 0.06) |  | -0.04  (-0.11, 0.04) |  | | -0.06  (-0.14, 0.01) |  |
| A level | 0.08  (0.00, 0.15) |  | -0.15  (-0.22, -0.07) |  | 0.04  (-0.04, 0.11) |  | -0.06  (-0.14, 0.01) |  | | -0.02  (-0.10, 0.05) |  |
| Degree | 0.08  (0.00, 0.17) |  | -0.15  (-0.23, -0.06) |  | 0.08  (0.00, 0.17) |  | -0.14  (-0.23, -0.06) |  | | -0.02  (-0.11, 0.07) |  |
| UVB protection score | 0.01  (0.00, 0.02) | 0.10 | 0.00  (-0.01, 0.01) | 0.98 | -0.01  (-0.02, 0.00) | 0.26 | 0.01  (0.00, 0.02) | 0.18 | | -0.01  (-0.02, 0.00) | 0.08 |
| Average h/day spent outdoors during summer | 0.06  (0.03, 0.08) | <0.001 | 0.02  (0.00, 0.05) | 0.08 | 0.01  (-0.01, 0.04) | 0.29 | 0.02  (-0.01, 0.04) | 0.14 | | -0.02  (-0.04, 0.01) | 0.12 |
| Family history of depression/schizophrenia | | |  |  |  |  |  |  | |  |  |
| None | -0.09  (-0.20, 0.01) | <0.001 | 0.05  (-0.16, 0.26) | 0.08 | 0.01  (-0.20, 0.22) | 0.79 | 0.01  (-0.20, 0.23) | <0.001 | | 0.01  (-0.20, 0.22) | 0.65 |
| Depression | -0.09  (-0.15, -0.03) |  | 0.06  (0.00, 0.11) |  | 0.00  (-0.06, 0.05) |  | 0.04  (-0.02, 0.10) |  | | 0.02  (-0.04, 0.07) |  |
| Schizophrenia | -0.22  (-0.43, -0.01) |  | 0.02  (-0.19, 0.23) |  | 0.10  (-0.11, 0.31) |  | -0.14  (-0.36, 0.07) |  | | 0.00  (-0.21, 0.21) |  |
| Puberty stage at serum measurement | |  |  |  |  |  |  |  | |  |  |
| 1 | reference | 0.009 | reference | 0.85 | reference | 0.08 | reference | 0.06 | | reference | 0.10 |
| 2 | -0.06  (-0.12, 0.00) |  | -0.01  (-0.07, 0.05) |  | 0.04  (-0.02, 0.10) |  | 0.02  (-0.04, 0.08) |  | | 0.04  (-0.02, 0.10) |  |
| 3 | -0.04  (-0.11, 0.03) |  | -0.02  (-0.09, 0.05) |  | 0.08  (0.01, 0.15) |  | 0.02  (-0.05, 0.09) |  | | 0.08  (0.02, 0.15) |  |
| 4-5 | -0.15  (-0.25, -0.04) |  | 0.02  (-0.08, 0.13) |  | 0.02  (-0.08, 0.13) |  | 0.13  (0.02, 0.24) |  | | 0.00  (-0.10, 0.11) |  |

**Online Supplementary Table 3.** Univariable associations between potential confounders, exposures and depressive symptoms

|  | Depressive symptoms (10.6 years) | | Depressive symptoms (13.8 years) | |
| --- | --- | --- | --- | --- |
|  | OR for category change per SD/category change (95%CI) | *P* | OR for category change per SD/category change (95%CI) | *P* |
| BMI (kg/m^2^) | 1.01 (1.00, 1.03) | 0.16 | 1.03 (1.01, 1.05) | 0.001 |
| WISC full IQ score at 8.5 years | 0.99 (0.99, 0.99) | <0.001 | 1.00 (1.00, 1.01) | 0.21 |
| Non-white ethnicity | 1.01 (0.82, 1.24) | 0.92 | 1.02 (0.81, 1.29) | 0.86 |
| Head of household social class | |  |  |  |
| i | 1.0 (reference) | 0.003 | 1.0 (reference) | 0.99 |
| ii | 1.12 (0.98, 1.28) |  | 0.94 (0.82, 1.08) |  |
| iii non-manual | 1.14 (0.98, 1.31) |  | 0.93 (0.80, 1.09) |  |
| iii manual | 1.27 (1.06, 1.52) |  | 0.97 (0.79, 1.19) |  |
| iv/v | 1.35 (1.06, 1.72) |  | 1.05 (0.79, 1.39) |  |
| Paternal education |  |  |  |  |
| None/CSE | 1.0 (reference) | 0.007 | 1.0 (reference) | 0.67 |
| Vocational | 1.06 (0.89, 1.28) |  | - 1. (0.82, 1.24) |  |
| O level | 0.78 (0.68, 0.89) |  | 0.96 (0.82, 1.12) |  |
| A level | 0.89 (0.78, 1.01) |  | 0.96 (0.83, 1.11) |  |
| Degree | 0.84 (0.73, 0.96) |  | 1.06 (0.91, 1.23) |  |
| Maternal education | |  |  |  |
| None/CSE | 1.0 (reference) | 0.003 | 1.0 (reference) | 0.27 |
| Vocational | 0.81 (0.67, 0.98) |  | 0.93 (0.75, 1.16) |  |
| O level | 0.82 (0.72, 0.95) |  | 0.96 (0.82, 1.13) |  |
| A level | 0.84 (0.72, 0.97) |  | 0.98 (0.83, 1.15) |  |
| Degree | 0.75 (0.63, 0.88) |  | 1.09 (0.91, 1.31) |  |
| UVB protection score | 1.00 (0.98, 1.02) | 0.71 | 0.99 (0.96, 1.01) | 0.20 |
| Average h/day spent outdoors during summer | 1.01 (0.97, 1.06) | 0.56 | 0.91 (0.87, 0.96) | <0.001 |
| Family history of depression/schizophrenia | |  |  |  |
| None | 1.0 (reference) | <0.001 | 1.0 (reference) | <0.001 |
| Depression | 1.28 (1.15, 1.43) |  | 1.43 (1.27, 1.62) |  |
| Schizophrenia | 0.95 (0.63, 1.42) |  | 1.24 (0.79, 1.95) |  |
| Puberty stage at serum measurement | |  |  |  |
| 1 | 1.0 (reference) | 0.34 | 1.0 (reference) | 0.36 |
| 2 | - 1. (0.91, 1.15) |  | 0.92 (0.81, 1.04) |  |
| 3 | 1.07 (0.93, 1.22) |  | 0.91 (0.78, 1.05) |  |
| 4-5 | - 1. (0.86, 1.31) |  | 1. (0.80, 1.25) |  |

**Online Supplementary Table 4. Prospective association of unadjusted 25(OH)D_3_ with depressive symptoms assessed by short Mood and Feelings Questionnaire at age 10.6 years (n=2759, exposures assessed at mean age 9.2 years) and age 13.8 years (n=2752, exposures assessed at mean age 9.8 years)**

| Outcome | OR^1^ for category change per doubling of exposure (95%CI) | | |
| --- | --- | --- | --- |
|  | Model 1^2^ | Model 2^3^ | Model 3^4^ |
| Depressive symptoms at age 10.6 years | 0.99 (0.94, 1.03) | 0.98 (0.93, 1.03) | 0.98 (0.93, 1.03) |
| Depressive symptoms at age 13.8 years | 0.90 (0.85, 0.94) | 0.90 (0.85, 0.95) | 0.89 (0.85, 0.94) |

^1^OR = Odds ratio

^2^Model 1 is unadjusted (25OHD_3_ is standardised for age and gender)

^3^Model 2 is adjusted for ethnicity, head of household social class, mothers and partners education, time spent outdoors during summer (age 8.5 years), UVB protection score, WISC IQ score at 8.5 years, BMI, family history of psychiatric problems and puberty stage

^4^Model 3 is adjusted for Model 2 plus serum concentrations of phosphate, albumin-adjusted calcium and parathyroid hormone)

**Online Supplementary Table 5. Prospective association of total 25(OH)D (25(OH)D_3_ + 25(OH)D_2)_ with depressive symptoms assessed by short Mood and Feelings Questionnaire at age 10.6 years (n=2759, exposures assessed at mean age 9.2 years) and age 13.8 years (n=2752, exposures assessed at mean age 9.8 years)**

| Outcome | OR^1^ for category change per doubling of exposure (95%CI) | | |
| --- | --- | --- | --- |
|  | Model 1^2^ | Model 2^3^ | Model 3^4^ |
| Depressive symptoms at age 10.6 years | 0.99 (0.94, 1.03) | 0.98 (0.93, 1.03) | 0.98 (0.93, 1.03) |
| Depressive symptoms at age 13.8 years | 0.90 (0.85, 0.94) | 0.90 (0.85, 0.95) | 0.89 (0.85, 0.94) |

^1^OR = Odds ratio

^2^Model 1 is unadjusted (25(OH)D is standardised for age and gender)

^3^Model 2 is adjusted for ethnicity, head of household social class, mothers and partners education, time spent outdoors during summer (age 8.5 years), UVB protection score, WISC IQ score at 8.5 years, BMI, family history of psychiatric problems and puberty stage

^4^Model 3 is adjusted for Model 2 plus serum concentrations of phosphate, albumin-adjusted calcium and parathyroid hormone)
